# Supplementary material for: GM1 Is Cytoprotective in GPR37-Expressing Cells and Downregulates Signaling
Source: Int J Mol Sci. 2021 Nov 27;22(23):12859. doi: 10.3390/ijms222312859 (PMC8657933; doi:10.3390/ijms222312859)
Supplement: Supplementary file 1 [file ijms-22-12859-s001.zip › ijms-1440934 supplementary.pdf]

Supplementary Information and Figures

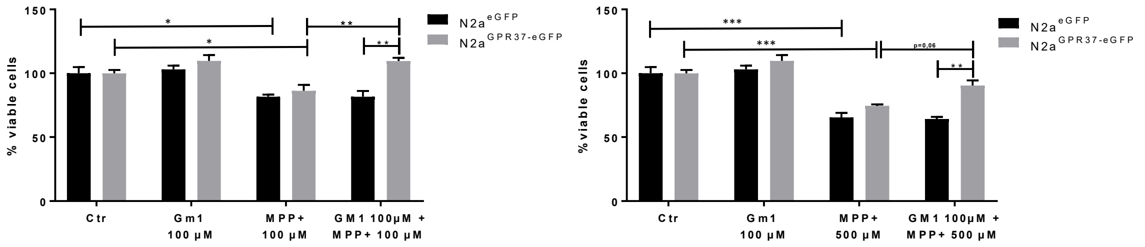

Supplementary Figure S1. GPR37-specific rescue with GM1 treatment (100 µM) against MPP+ treatments of different concentrations. GPR37-specific rescue of GM1 pretreatment (100 µM) against MPP+ treatments of different concentrations in stable $N2a^{GPR37\text{-}eGFP}$ compared to $N2a^{eGFP}$ after 24 h measured by rezasurin assay. n=3 * $p<0.05$ ** $p<0.01$, *** $p<0.001$ (2-way ANOVA, Tukey posthoc test).

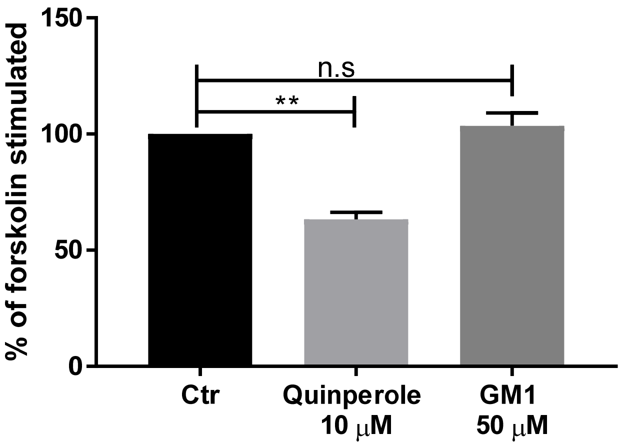

Supplementary Figure S2. Positive control in signaling experiments. Signaling from transiently transfected HEK293T with D2R-tdTomato show expected decrease in cAMP when stimulated with 10 µM quinpirole. GM1 does not change cAMP level. n=3, ** $p<0.01$, (Kruskal-Wallis test followed by Dunn's test for multiple comparisons).

Homology modeling

GPR37 structure was predicted using MODELLER[1] with the crystal structure of the Endothelin B receptor (PDB ID: 5GLH) used as a template. Briefly, the sequences were aligned manually using Jalview[2] followed by the generation of 100 models using MODELLER, which were ranked based on the DOPE score [3]. The top models were then evaluated manually and refined. The predicted active structure of 5HT1A was obtained from GPCRdb and aligned to the top GPR37 model using Pymol. Similarly, inactive and active predicted structures of GPR37 were obtained from GPCRdb[4], aligned and compared with the active or inactive predicted model of the 5HT1A receptor.

A) B)

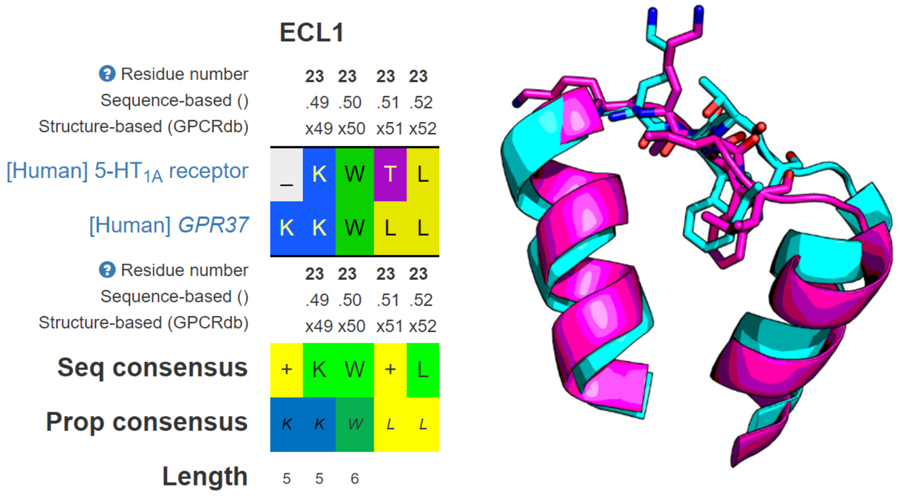

Supplementary Figure S3. Homology modeling method and figure. Sequence alignment from GPCRdb[4, 5] showing conserved lysine and tryptophan residues, claimed to be important for GM1 binding to 5HT1A (A). Model of inactive states with GPR37 in magenta and 5HT1A in turquoise (B).

References

1. Webb B, Sali A. Comparative Protein Structure Modeling Using MODELLER. Curr Protoc Bioinformatics 2016;54:5.6.1-5.6.37.
2. Waterhouse AM, Procter JB, Martin DM, Clamp M, Barton GJ. Jalview Version 2--a multiple sequence alignment editor and analysis workbench. Bioinformatics 2009;25(9):1189-1191.
3. Shen MY, Sali A. Statistical potential for assessment and prediction of protein structures. Protein Sci 2006;15(11):2507-2524.
4. Pándy-Szekeres G, Munk C, Tsonkov TM, et al. GPCRdb in 2018: adding GPCR structure models and ligands. Nucleic Acids Res 2018;46(D1):D440-D446.
5. Isberg V, de Graaf C, Bortolato A, et al. Generic GPCR residue numbers - aligning topology maps while minding the gaps. Trends Pharmacol Sci 2015;36(1):22-31.
